# Supplementary material for: Thymosin β4 preserves vascular smooth muscle phenotype in atherosclerosis via regulation of low density lipoprotein related protein 1 (LRP1)
Source: Int Immunopharmacol. 2023 Feb;115:None. doi: 10.1016/j.intimp.2023.109702 (PMC10666903; doi:10.1016/j.intimp.2023.109702)
Supplement: Supplementary data 1 [file mmc1.pdf]

Thymosin β4 preserves vascular smooth muscle phenotype in atherosclerosis via regulation of Low Density Lipoprotein Related Protein 1 (LRP1).

Sonali Munshaw[1], Andia N. Redpath[1], Benjamin T. Pike[1] & Nicola Smart[1]*.

1. Burdon Sanderson Cardiac Science Centre, Department of Physiology, Anatomy & Genetics, University of Oxford, Sherrington Building, South Parks Road, Oxford OX1 3PT, UK.

SUPPLEMENTAL MATERIAL

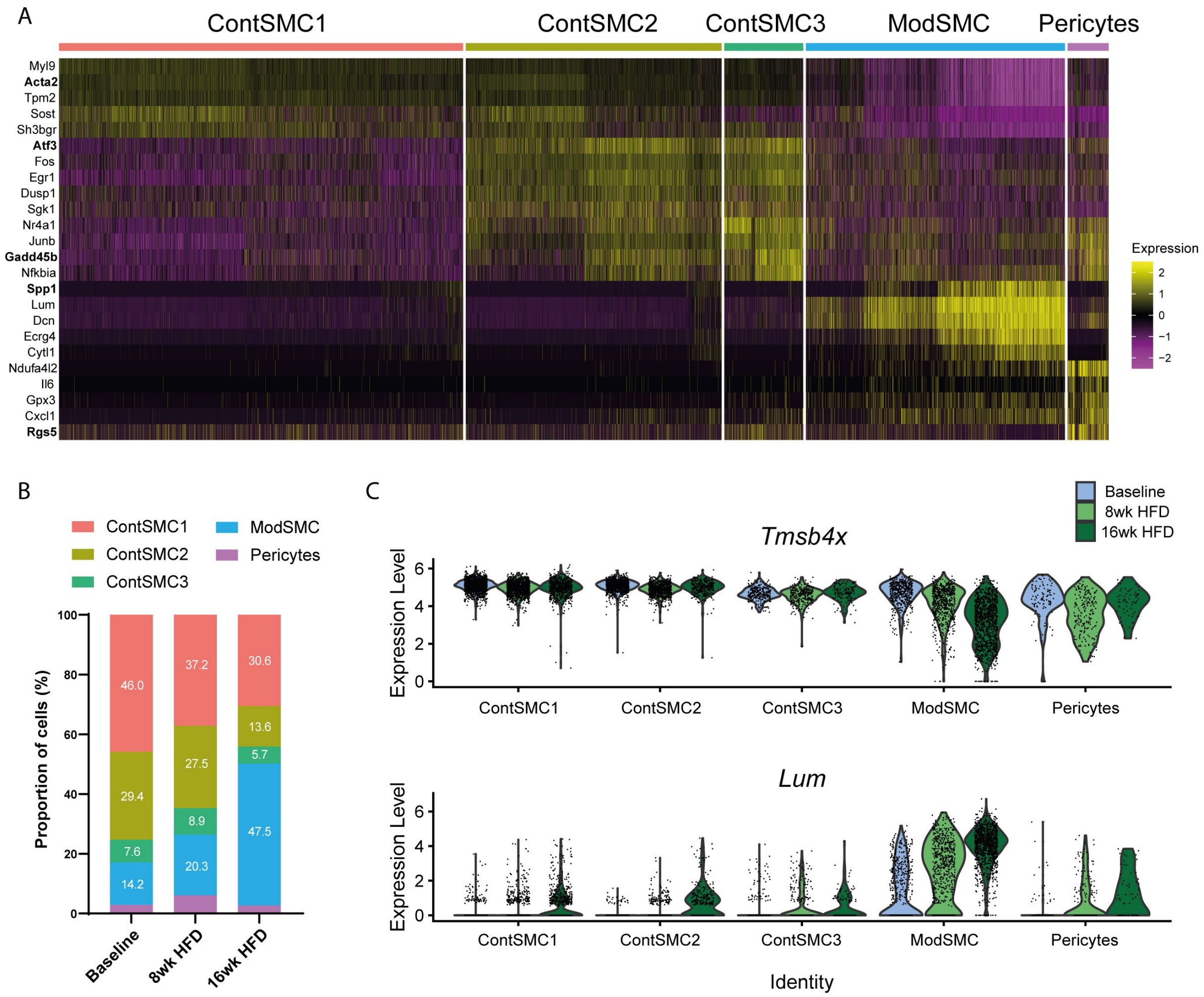

Online Figure I. scRNA-seq of VSMCs in atherosclerosis.
A: Computational analysis of scRNA-seq of lineage traced aortic VSMCs from ApoE$^{-/-}$ mice (derived from[23]) identified 5 VSMC subpopulations, as shown in Figure 1. Heatmap illustrating the top 5 marker genes and their expression levels to characterise each cluster. **B**: Stacked bar plot depicting the proportions of each VSMC subpopulation at each time point, to illustrate VSMC modulation in atherosclerosis. **C**: Violin plots to demonstrate changes in *Tmsb4x* and *Lumican* expression across the five VSMC subpopulations over the disease time course.

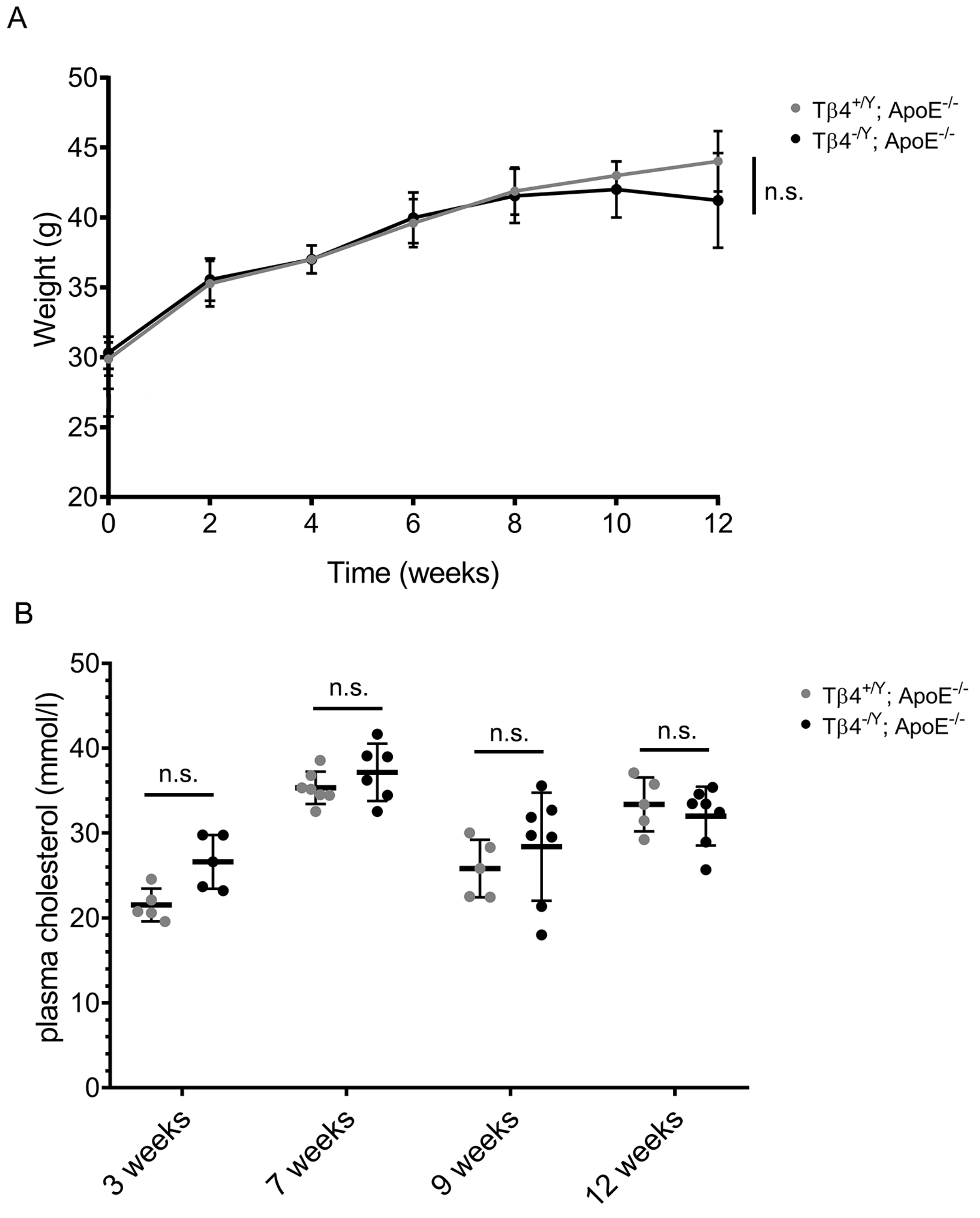

Online Figure II. Tβ4$^{+/Y}$; ApoE$^{-/-}$ and Tβ4$^{-/Y}$; ApoE$^{-/-}$ do not show differences in weight gain or cholesterol levels after western diet. Comparison of weight gain (**A**) and plasma cholesterol levels (**B**) in Tβ4$^{+/Y}$; ApoE$^{-/-}$ and Tβ4$^{-/Y}$; ApoE$^{-/-}$ mice over the time course of the high fat diet feeding regime. Data are presented as mean ± SD, with n=11 in **A**. Each data point in **B** represents an individual animal. Significance was calculated using two-way ANOVA with Bonferroni correction for multiple comparisons (**A**) and one-Way ANOVA with Tukey's multiple comparison tests (**B**). n.s. = not significant; ***: p≤0.001

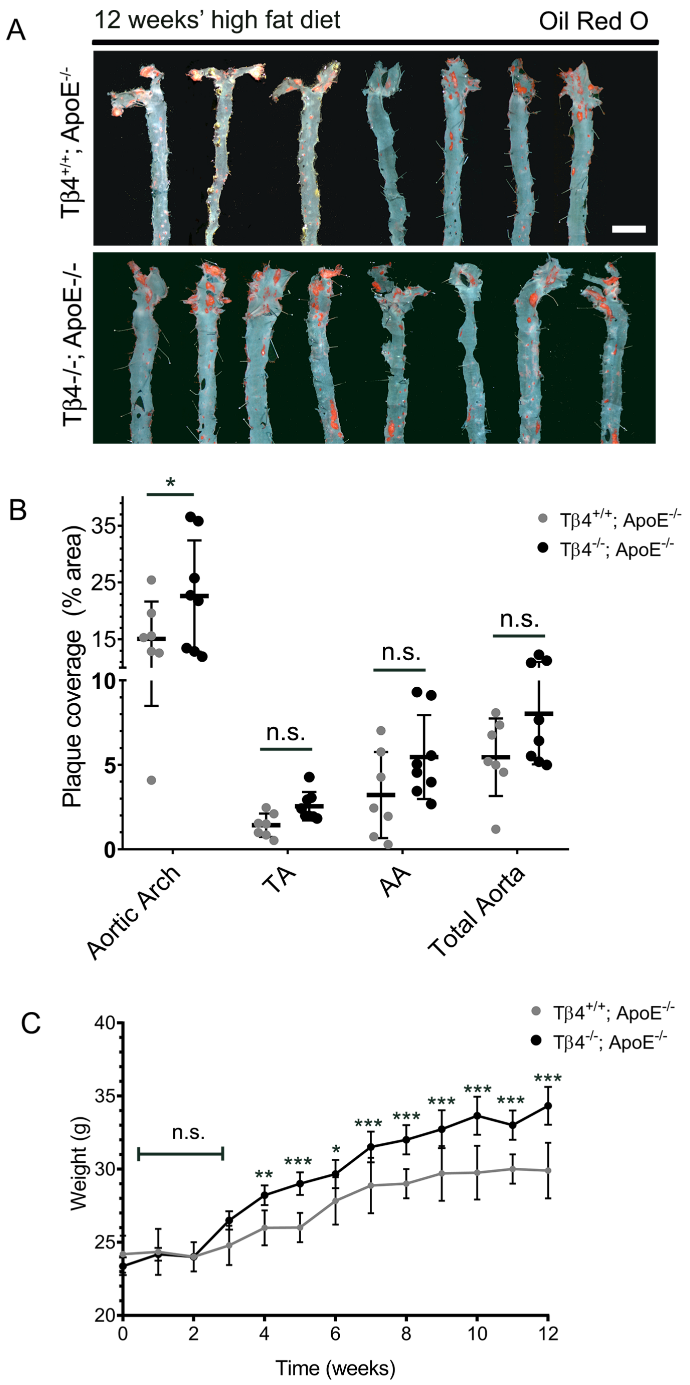

Online Figure III. Increased predisposition to atherosclerotic plaque formation was also observed in female Tβ4$^{-/-}$; ApoE$^{-/-}$ mice. *En face* aorta preparations and oil red O staining to visualize plaques in female mice fed Western diet for 12 weeks (**A**). Quantification of plaque coverage in the aortic arch, thoracic aorta (TA), abdominal aorta (AA) and total aorta (**B**). Weight gain was significantly increased in female Tβ4$^{-/-}$; ApoE$^{-/-}$, compared with Tβ4$^{-/-}$; ApoE$^{-/-}$ mice from 4-12 weeks' high fat diet feeding (**C**). Data are presented as mean ± SD, with each data point representing an individual animal (**B**) and n=8 (**C**). Significance was calculated using one-way ANOVA with Tukey's multiple comparison tests (**B**) and two-way ANOVA with Dunnett's post hoc tests (**C**). n.s.= not significant; *p ≤ 0.05; **p≤0.01; ***: p≤0.001.
